# HM015k, a Novel Silybin Derivative, Multi-Targets Metastatic Ovarian Cancer Cells and Is Safe in Zebrafish Toxicity Studies

**DOI:** 10.3389/fphar.2017.00498

**Published:** 2017-08-02

**Authors:** Haneen Amawi, Noor A. Hussein, Chandrabose Karthikeyan, Elangovan Manivannan, Alexander Wisner, Frederick E. Williams, Temesgen Samuel, Piyush Trivedi, Charles R. Ashby, Amit K. Tiwari

**Affiliations:** ^1^Department of Pharmacology and Experimental Therapeutics, College of Pharmacy and Pharmaceutical Sciences, University of Toledo Toledo, OH, United States; ^2^School of Pharmaceutical Sciences, Rajiv Gandhi Proudyogiki Vishwavidyalaya Bhopal, India; ^3^School of Pharmacy, Devi Ahilya Vishwavidyalaya Indore, India; ^4^Department of Pathobiology, School of Veterinary Medicine, Tuskegee University Tuskegee, AL, United States; ^5^Pharmaceutical Sciences, College of Pharmacy, St. John's University Queens New York, NY, United States

**Keywords:** ovarian cancer, silybin, silymarin, metastasis, apoptosis, epithelial-mesenchymal transition, zebrafish, drug discovery

## Abstract

This study was designed to determine the *in vitro* mechanisms by which the novel silybin derivative, (E)-3-(3-(benzyloxy) phenyl)-1-(4-hydroxyphenyl)prop-2-en-1-one (**HM015k** or **15k**), produces its anticancer efficacy in ovarian cancer cells. Compound **15k** induced apoptosis in ovarian cancer cells in a time-dependent manner by significantly upregulating the expression of Bax and Bak and downregulating the expression of Bcl-2. Interestingly, **15k** induced the cleavage of Bax p21 into its more efficacious cleaved form, Bax p18. In addition, caspase 3 and caspase 9 were cleaved to their active forms, inducing the cleavage of poly ADP ribose polymerase (PARP) and β-catenin. Furthermore, in OV2008 cells, **15k** induced significant cleavage in nuclear β-catenin to primarily inactive fragments of lower molecular weight. Furthermore, **15k** reversed the metastatic potential of OV2008 cells by inhibiting their migration and invasiveness. The mesenchymal phenotype in OV2008 was reversed by **15k**, causing cells to be rounder with epithelial—like phenotypes. The **15k**-induced reversal was further confirmed by significant upregulation of the E-cadherin expression, an epithelial marker, while N-cadherin, a mesenchymal marker, was downregulated in OV2008 cells. Compound **15k** inhibited the expression of the oncogenic c-Myc protein, downregulated proteins DVL3 and DVL2 and significantly upregulated cyclin B1. Also, **15k** significantly downregulated the expression levels of ABCG2 and ABCB1 transporters in resistant ABCG2 overexpressing H460/MX20 and resistant ABCB1 overexpressing MDCK/MDR1 cells, respectively. Finally, **15k** was safe in zebrafish *in vivo* model at concentrations up to 10 μM and induced no major toxicities in cardiac, morphology and swimming position parameters. Overall, **15k** is a multi-targeted inhibitor with efficacy against metastatic and resistant ovarian cancer. Future *in vivo* studies will be conducted to determine the efficacy of **15k** in tumor-bearing animals.

## Introduction

Ovarian cancer is one of most lethal malignancies in women and is responsible for 5% of all the cancer deaths in women (American Cancer Society, 2017). There has been a steady decline in the incidence of ovarian cancer since the mid-1970s (American Cancer Society, 2017). However, many patients are still diagnosed in advanced stages (III-IV) of the disease (60%), significantly decreasing their survival rates (≈46%) (Goodman et al., 2003; American Cancer Society, 2017). Unlike other epithelial cancer cells, ovarian cancer cells can disseminate directly to the peritoneum cavity due to the absence of anatomical barriers (Armstrong, 2002; Sehouli et al., 2009). In addition, recent data indicates that the majority of patients will relapse despite a satisfactory response to the initial treatment (Bagnato and Rosanò, 2012; Luvero et al., 2014). The resistance of ovarian cancer cells to treatment can result from: DNA repair, high glutathione content, overexpression of ABCB1 or ABCG2 transporters, and epithelial-mesenchymal transition (EMT), among others (Ozols et al., 2003; Ahmed et al., 2007; Galluzzi et al., 2012; Varga et al., 2014). Recently, ovarian cancer was classified into 5 different types: (1) high-grade serous carcinoma; (2) low-grade serous carcinoma; (3) clear cell carcinoma; (4) endometrioid carcinoma and (5) mucinous carcinoma (Kurman et al., 2014). The different types of ovarian cancer vary in their histopathology, clinical outcomes and molecular pathways.

The Wnt/β-catenin signaling pathway has been reported to be dysregulated in several types of ovarian cancer (Wright et al., 1999; Wu et al., 2001). Recent studies also showed that epithelial—mesenchymal transition (EMT) plays a role in the progression and metastasis of ovarian carcinomas (Ahmed et al., 2010; Vergara et al., 2010). During EMT, cells transition from the epithelial to mesenchymal state, characterized by the loss of cell to cell adhesion, cell polarity and differentiation properties and become invasive and motile, with inhibition of apoptotic pathways and induction of metastasis (Geiger and Peeper, 2009). Currently, a platinum-taxane combination that may be administered abdominally, can be used for certain cases of ovarian cancer (Vaughan et al., 2011; Schwab et al., 2014). However, relatively few patients receive this treatment due to the development of severe adverse effects and toxicities, including bleeding, infections, and neurotoxicitiy, and others (Fotopoulou, 2014). Additionally, although the tumors of epithelial ovarian cancer appear to shrink after the initial cycles of treatment, the malignant cells can regrow and more than 90% of patients have disease recurrence (Santin et al., 2000). Multidrug resistance to these treatments, e.g., overexpression of ABC transporters, is another major obstacle that limits their beneficial actions (Tiwari et al., 2011, 2013). Consequently, there is an essential need to develop and design new compounds with novel, multi-targeted mechanisms of action that are efficacious against metastatic, resistant and aggressive ovarian cancer and have an acceptable toxicity profile.

**Figure 1:**
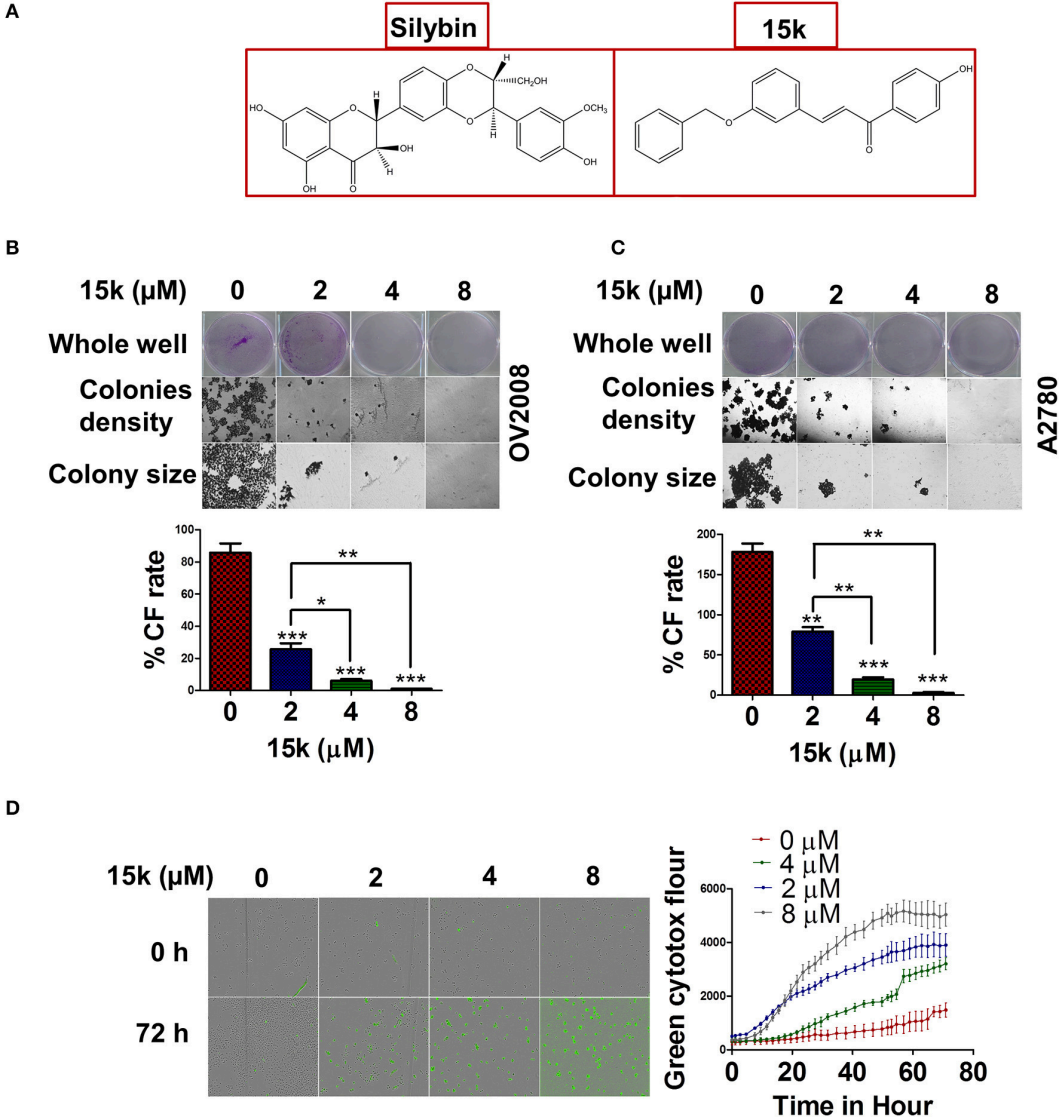
15k effect on colony formation rate and viability of ovarian cancer cells **(A)** The chemical structures of silybin A and **15k (B,C)** Representative images of the whole well, the densities of the colonies formed (10x) and the colony size (20x) of OV2008 and A2780, respectively, after incubation with **15k** (0, 2, 4, and 8 μM). The colony formation rate (%CF rate) is shown under each cell type in the graph. The results are presented as the means ± SD of three independent experiments. ^*^p < 0.05, ^**^p < 0.01, ^***^p < 0.001. **(D)** The real time green cytotox fluorescent reagent (IncuCyte) indicating the number of dead OV2008 cells over time after incubation with **15k** (0, 2, 4, and 8 μM). The data are presented as images showing the fluorescence level at the 0 and 72 h time points. In addition, a detailed time curve quantitatively summarizing the results at each time point is shown where fluor is for fluorescence. The data are presented as the means ± SEM of three independent studies.

In the last decade, numerous studies have reported that the natural compound silybin, found in the dried fruits of milk thistle plant (*Silybum marianum*), has anticancer efficacy *in vitro* and *in vivo* (Hoh et al., 2006; Deep and Agarwal, 2010; Flaig et al., 2010). Silybin (Figure 1A) was shown to significantly inhibit proliferation and metastasis via several targets in ovarian cancer cells. Silybin also significantly inhibits the Wnt/β-catenin/ EMT signaling in several cancer models (Kaur et al., 2010; Lu et al., 2012; Wu et al., 2013; Eo et al., 2016). However, silybin is poorly absorbed and has a low bioavailability (<0.95% in rats) as it is a substrate of drug metabolizing enzymes (especially phase II) (Lorenz et al., 1984; Barzaghi et al., 1990; Wen et al., 2008; Kren et al., 2013). Consequently, we have been conducting studies to find analogs of silybin with a desirable pharmacokinetic profile and significant anticancer efficacy. We previously reported the synthesis and development of 11 novel silybin derivatives (**HM015a–HM015k**) or (**15a–15k**) (Manivannan et al., 2017). The compounds were screened against breast (MCF-7, MDAMB-231, ZR-75-1, BT-20), prostate (DU-145), pancreatic (PANC1) and ovarian (OV2008, A2780) cancer cell lines. They were also screened in normal cell lines, including epithelial colon cells (CRL1459) and Chinese hamster ovary cells (CHO). The initial cytotoxic screening indicated that several silybin derivatives had significant anticancer efficacy (Manivannan et al., 2017). One of the compounds, **HM015k** or **15k**, (Figure 1A), had significant anticancer efficacy (IC_50_ < 1 μM) in ovarian cancer cells (IC_50_ = 0.8 ± 1 μM for OV2008 and 1 ± 0.1 μM for A2780) (Manivannan et al., 2017). Compound **15k** was significantly more efficacious in inhibiting the proliferation of ovarian cancer cells compared to other cancer cells lines and normal epithelial cells (IC_50_ = 8.5 ± 0.7 μM for CRL1459 and 8.1 ± 1.2 μM for CHO and thus, was 10-fold more selective for cancer vs. normal cell lines). Furthermore, **15k** produced cell cycle arrest at the sub-G1 phase, induced apoptosis and, inhibited tubulin protein expression and function. The present study was designed and conducted to elucidate the cellular and molecular pharmacological mechanisms of **15k**, its effect on metastasis, invasiveness, and recurrence in ovarian cancer cell lines, as well as its safety in larval zebrafish model.

## Materials and methods

### Chemicals and reagents

The antibodies used in this experiment, including primary (Bak, Bax, Bcl-2, PARP, cleaved caspase 3, cleaved caspase 9, E-cadherin, N-cadherin, DVL-2, DVL-3, c-Myc, Cyclin B1, β-catenin, Histone 3 and β-actin) and secondary (secondary anti-mouse, secondary anti-mouse) antibodies, and anti-rabbit IgG (R) Alexa Fluor® 488 molecular probe and Anti-mouse IgG (R) Alexa Fluor® 488 molecular probe were purchased from Cell Signaling Technology (Danvers, MA, USA). Propidium iodide (PI) dye was purchased Life Technologies (Eugene, Oregon, USA). Fluoroshield mounting medium, containing 4',6-diamidino-2-phenylindole dihydrochloride (DAPI), was purchased from Abcam (Cambridge, MA, USA). Crystal violet dye was purchased from Sigma-Aldrich (St. Louis, MO, USA). Dulbecco's Modified Eagle Medium (DMEM) was purchased from GE Healthcare Life Sciences and HyClone Laboratories (Logan, Utah, USA). Green cytotox, Annexin red and caspase 3/7 IncuCyte reagents were purchased from Essen BioScience (Ann Arbor, MI 48108, USA). The 6.5 mm diameter inserts, with 8.0 μM pore size polycarbonate membrane for 24 well plates, were purchased from Corning Incorporated (One Riverfront Plaza Corning, NY 14831, USA). Growth-factor-reduced matrigel was purchased from BD Biosciences (Ontario, Canada).

### Cell culture

Ovarian (A2789, OV2008) cancer cell lines were grown as adherent monolayers in flasks with Dulbecco's modified Eagle medium (DMEM), supplemented with 10% fetal bovine serum (FBS), in a humidified incubator with 5% CO_2_ at 37°C. In addition, the ABCG2 - overexpressing H460/MX20 and the ABCB1 overexpressing MDCK/MDR1 cells were also grown for immunofluorescence studies. Unless otherwise specified, before any incubation with the test compound, the cells were allowed to grow for 24 h in DMEM to reach 30–40% confluency, then incubated with **15k** for additional 24 h and harvested with 0.25% trypsin, 2.21 mM EDTA, 1X from Corning (Corning, NY 14831USA).

### Colony formation assay

The colony formation assay was done to measure the rate and formation of colonies in ovarian cancer cells formed in the presence or absence of compound **15k**. Initially, cells were seeded as 250,000 cells/well and then were incubated with 0, 2, 4, or 8 μM of **15k**. The cells were harvested, counted and reseeded as 500 cells/well (1 ml in each well) in new 6-well plate, with complete medium, for 10 days. The medium was changed every 2 days to prevent cell starvation. The colonies were fixed with absolute methanol at room temperature for 30 min. Finally, 0.1% crystal violet dye (Sigma, USA) was added to each well to stain the cells for another 30 min. The stained, fixed colonies were viewed under an EVOS microscope (Themo Fisher Scientific, Wayne, MI, USA) and counted. The colony formation rate was calculated as follows: the number of colonies formed divided by initial number of seeded cells (500) × 100%.

### Time-dependent cytotoxicity assays

The Incucyte green cytotox dye was used for real time quantification of non-viable OV2008 cells. The fluorescent dye can only penetrate into cells with non-intact membranes, i.e., non-viable or dead cells. The dye binds to nucleic acids and emits green fluorescence at an excitation maximum of 491 nm and emission maximum of 509 nm. After seeding and incubating with **15k**, the cytotox green reagent was added at a final concentration of 0.25 μM. Subsequently, the cells were immediately placed in the IncuCyte Zoom live cell imaging apparatus and pictures were taken every 2 h for up to 72 h and analyzed using the integrated software (IncuCyte ZOOM version 2016A, Essen BioScience, Ann Arbor, MI 48108, USA).

### Reactive oxygen species (ROS) detection

To detect ROS generation, we used the 2′,7′-dichlorofluorescin (H_2_DCFDA) reagent as previously described (Amawi et al., in press). Initially, a 5 mM stock solution was prepared using DMSO. PBS was then used to prepare the 3 μM working solution. OV2008 cells were seeded in 6 well plates and incubated with 0, 1, 2, and 4 μM of **15k** for 24 or 48 h. The working solution of H_2_DCFDA was added to each well and the cells were incubated for an additional 30 min at 37°C. PBS was used to wash (3 times) the cells from the traces of the dye working solution. Finally, the fluorescence magnitude of the oxidized form of the dye (DCF) was determined using EVOS digital fluorescent microscope at 40x.

### Time-dependent apoptosis induction

OV2008 and A2780 cells were seeded in 96 well plates at a density of 1 × 10^3^ cell/well. Twenty four hours later, the cells were incubated with **15k** (0, 2, 4, and 8 μM) for 72 h. The apoptosis detecting reagents (caspase 3/7 reagent or annexin red reagent) were added to the cells immediately after 15 k and were incubated for up to 72 h at 37°C. Fluorescence was determined every 2 h using a live cell imaging system (IncuCyte Zoom) from Essen Bioscience (Ann Arbor, MI, USA).

### Determination of the *in vitro* Anti-metastatic efficacy of 15k

#### Wound healing assay

OV2008 cells were seeded and incubated until they reached 100% confluence and formed a complete monolayer in a 6-well plate. Subsequently, a wound was created by scratching the monolayer with a 200 μl sterile pipette tip. The floating cells were washed with sterile PBS making the wound area clear and empty. Immediately following this procedure, compound **15k** was added at different concentrations (0, 1, 2, and 4 μM) to the cell culture media to determine its efficacy in preventing wound closure (i.e., reverse migration). Finally, pictures were taken at different time points (0, 6, 18, 36, and 48 h) using an EVOS microscope system to detect the closure of the wound over time in cells incubated with **15k** or vehicle. Image J software (NIH, Bethesda, Maryland, USA) was used to determine the area of the wounds at different time points (the percentage closure).

#### Transwell migration assay

A 24 well plate with 24 well inserts of 8 μm pore size was used to determine cancer cell migration and invasive potential. The inserts were pre-coated with growth-factor-reduced matrigel (1:40) and added to each well, forming two chambers (upper and lower). The lower chambers were filled with cell-free DMEM and OV2008 cells were added to the upper chamber as 200 μl suspensions. After 1 h, **15k** was added and the cells were incubated for 24 h. Cotton swabs were used to remove the non-migrated or stationary cells from the inside of the upper chamber. The migrated cells were fixed with methanol and dyed with 0.1% crystal violet dye and visualized with an EVOS microscope. The effect of **15k** or vehicle was determined by counting the cells in the field of view.

### Cell lysis and western blot analysis

The lysis of cells was done in two stages to obtain nuclear and cytosolic proteins as previously described (Alhadidi and Shah, 2017). Both cytosolic and nuclear proteins were loaded in an acrylamide SDS-PAGE gel for protein separation. The separated proteins were transferred to a PVDF membrane to start the detection of the level of protein. The PVDF membrane was then incubated over night with primary antibodies against Bak (1;1,000), Bax (1;1,000), Bcl-2 (1;1,000), caspase 9 (1:5,000), caspase 3 (1;5,000), PARP (1;5,000), N-cadherin (1;5,000), E-cadherin (1;5,000), c-Myc (1;4,000), DVL3 (1;5,000), DVL2 (1;3,000), cyclin B1 (1;1,000), β-catenin (1;5,000), β-actin (1;5,000), or histone (1;4,000) in 5% milk at 4°C. Horseradish peroxidase-labeled (HRP) anti-rabbit or anti-mouse secondary antibodies (1:5,000 dilution) were added the next day for an additional 1 h. A ChemiDoc™ MP System imaging system from Bio-Rad (Hercules, California, USA). Blots were used to obtain the protein bands, followed by quantification of the bands using image J software (NIH, Bethesda, Maryland, USA). All of the data were expressed as the ratio of β-actin or histone.

### Immunofluorescence

OV2008 cells were seeded at a density of 4 × 10^4^ cell/well on culture cover glass inserted in 6 well plates and allowed to grow overnight. Next, the cells were incubated with **15k** (0, 2, and 4 μM) for an additional 24 h at 37°C. The incubated cells were fixed with 4% paraformaldehyde, followed by permeabilization with 0.3% of Triton X100 in PBS for 25 min. Subsequently, the cells were blocked with 5% BSA in PBS for at least 30 min, followed by the addition of the appropriate primary antibodies (β-catenin E-cadherin) (1:400, 1:200) for OV2008 cells, ABCG2 for H460/MX20 (1: 400) and ABCB1 for the MDCK/MDR1 (1:400) cell line (Hussein et al., 2017). Secondary antibody (fluorescent) was then added (1:700) for 1 h. Finally, DAPI or PI was added to each slide to stain the nucleus of each cell. The slides were allowed to dry for at least 1 h and the proteins of interest were detected under a fluorescent microscope (EVOS cell imaging system). The fluorescence/cell was quantified using the Image J software.

### Zebrafish larval toxicity studies

Wild-type adult zebrafish (Danio rerio) were housed in a light- and temperature-controlled facility with 14:10-h light–dark photoperiod, at a pH of 7.2 and fed on live brine shrimp 2 times a day and flake food once daily. Embryos were produced by spawning adult fish using a hatch box and the embryo medium was maintained at 28°C. Larvae were collected and used for the experiments. The 5 day post-fertilization larvae were placed in 24 well plates at a count of 5 fish/well and **15k** was added in the fish water at 8 different concentrations (0, 0.3, 1, 3, 6, 10, 30, and 100 μM). Both visual observation and images from an EVOS digital microscope (4x) were used to detect toxicities in larval zebrafish up to 48 hpe (hour post exposure). Fish were first observed for viability, where death was defined as the absence of a heartbeat (acute toxic dose). Other indications of toxicity included swim position (loss of dorsoventral balance as a sign of toxicity), and morphological abnormalities including malformations, body length, curved tail, and swim bladder inflation (swim BI) level. Finally, indices of cardiac toxicity included heart rate (HR) and regularity, as well as pericardial swelling or edema. The University of Toledo Institutional Animal Care and Use Committee approved the zebrafish studies (105414–MDeG).

### Statistical analysis

All of the experiments were done in triplicate and the data were statistically analyzed using either a one-way or two-way ANOVA. Specifically, the cell cycle assay, MitoTracker Red and Alexa Fluor 488 annexin V assay for apoptosis and wound healing assay data were analyzed by a two-way ANOVA, followed by Bonferroni's *post-hoc* analysis. The data from the colony formation assay, Western blots, DAPI staining, immunofluorescence, trans-well migration and the **15k** IC_50_ values for the different cells lines were analyzed by one-way ANOVA, followed by Tukey's *post-hoc* analysis. The results were represented as the mean ± the standard deviation (SD). The *a priori* significance level was *p* < 0.05.

## Results

### 15k significantly inhibits OV2008 cells viability and colony formation

Previous MTT cytotoxic data for OV2008 and A2780 cells indicated an IC_50_ of <1 μM for both cell lines (Manivannan et al., 2017). In this study, we also determined the effect of **15k** on colony formation in OV2008 and A2780 cells. Compound **15k** significantly inhibited (2, 4, or 8 μM) the colony formation rate in OV2008 cells compared to cells incubated with vehicle (*p* < 0.001 for all the three concentrations) (Figure 1B). Furthermore, the inhibition of colony formation produced by 4 and 8 μM of **15k** was significantly greater than that of 2 μM of **15k** (*p* < 0.05 for 4 μM compared to 2 μM and *p* < 0.01 for 8 μM compared to 2 μM, Figure 1B). Similarly, in A2780 cells, **15k** significantly inhibited colony formation (Figure 1C). At 2, 4, and 8 μM, **15k** significantly decreased the number of colonies formed (*p* < 0.01 for 2 μM and *p* < 0.001 for both 4 and 8 μM), compared A2780 cells incubated with vehicle (Figure 1C). It should be noted that in addition to the reduction in the number and the rate of the colonies formed, **15k** also significantly reduced the size of these colonies (Figures 1B,C).

Cytotoxicity was detected over time using the IncuCyte green cytotox reagent. As seen in the graph in Figure 1D, the number of fluorescent dead cells following incubation with vehicle, was low over time, even after 3 days of incubation, indicating that most of the cells are alive and viable (Figure 1D). However, 2 μM of **15k** significantly increased green fluorescence compared to cells incubated with vehicle for 24 h (Figure 1D). Compound **15k**, at 4 and 8 μM, significantly increased the number of dead cells at earlier time points (10–15 h) compared to 2 μM of **15k** (Figure 1D).

### 15k induces oxidative stress in HCT116, OV2008, and A2780 cells

**Figure 2:**
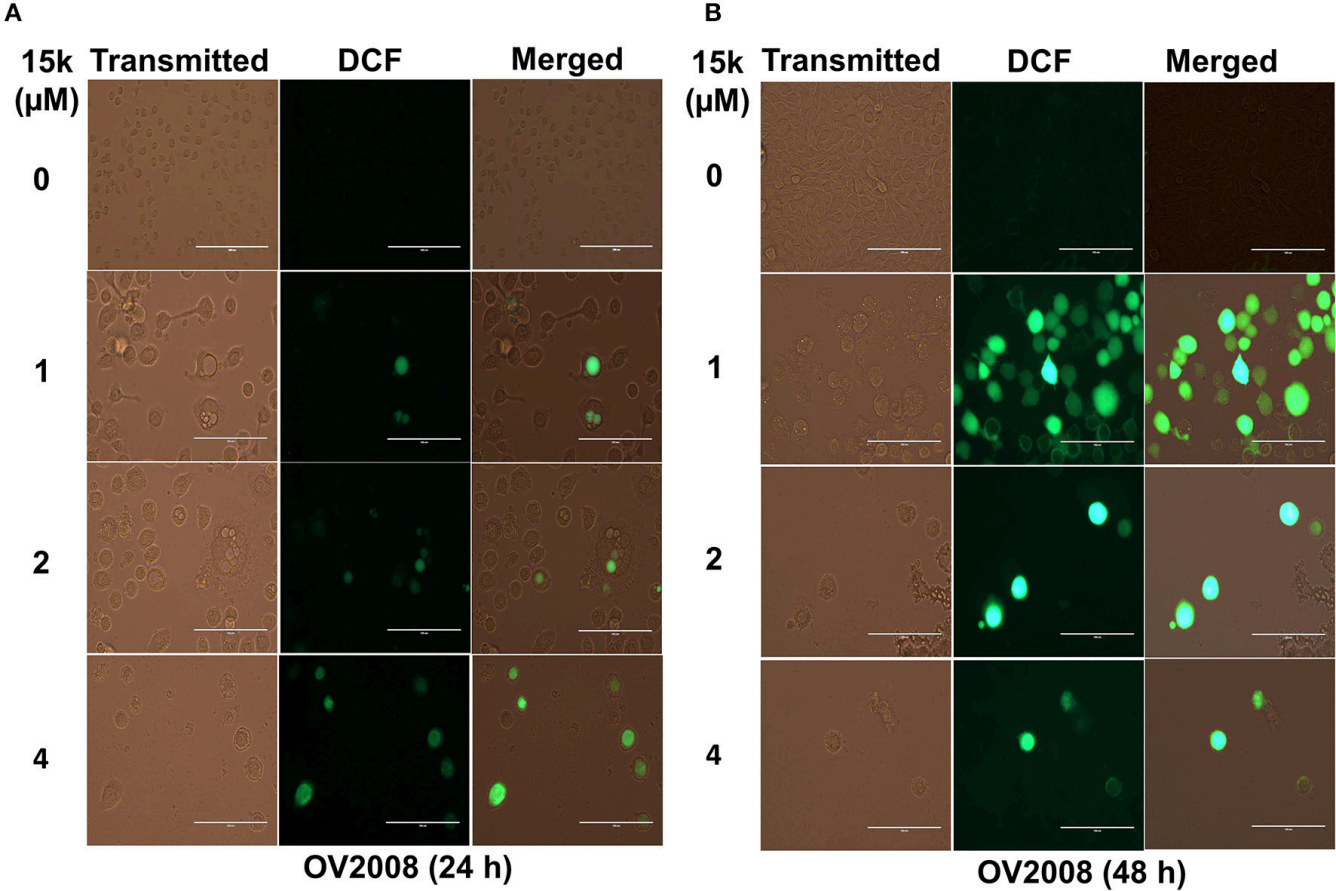
15k induces the generation of ROS in OV2008 cells; **(A,B)** Representative images of the DCF levels of fluorescence after incubation with 1, 2, and 4 μM of **15k** for 24 and 48 h compared to control, respectively. The experiments were repeated in triplicate.

The effect of **15k** on the generation of ROS was determined using the H_2_DCFDA stain. In this assay, cellular esterases cleave the nonfluorescent H_2_DCFDA to H_2_DCF by removing the lipophilic moiety (a diacetate group). Subsequently, H_2_DCF is oxidized by ROS to fluorescent DCF and the ROS levels were quantified based on the fluorescence level detected by EVOS. DCF fluorescence was significantly higher in ovarian cancer cells incubated with **15k** (Figure 2). The incubation of OV2008 with 1μM of **15k** for 24 h significantly increased the levels of ROS in some of the cells. However, at higher concentrations (2 and 4 μM), ROS levels were significantly greater than that measured after incubation with 1 μM of 15k (*p* < 0.05 for 1 and 2 μM and *p* < 0.01 for 4 μM (Figure 2A). In contrast, after 48 h of incubation with 1 μM of 15k, most of the cells expressed green fluorescence at a higher intensity (*p* < 0.001) compared to 24 h of incubation, indicating a significant increase in ROS). At 2 and 4 μM, most of the cells were dead and detached from the plate, as seen in the phase contrast images (Figure 2B). The remaining cells expressed the same or a higher level of fluorescence at 2 and 4 μM of 15k compared to 1 μM (*P* < 0.001 for 2 and 4 μM, Figure 2B).

### 15k induces apoptosis in a concentration- and time - dependent manner by activating the intrinsic apoptotic pathway

**Figure 3:**
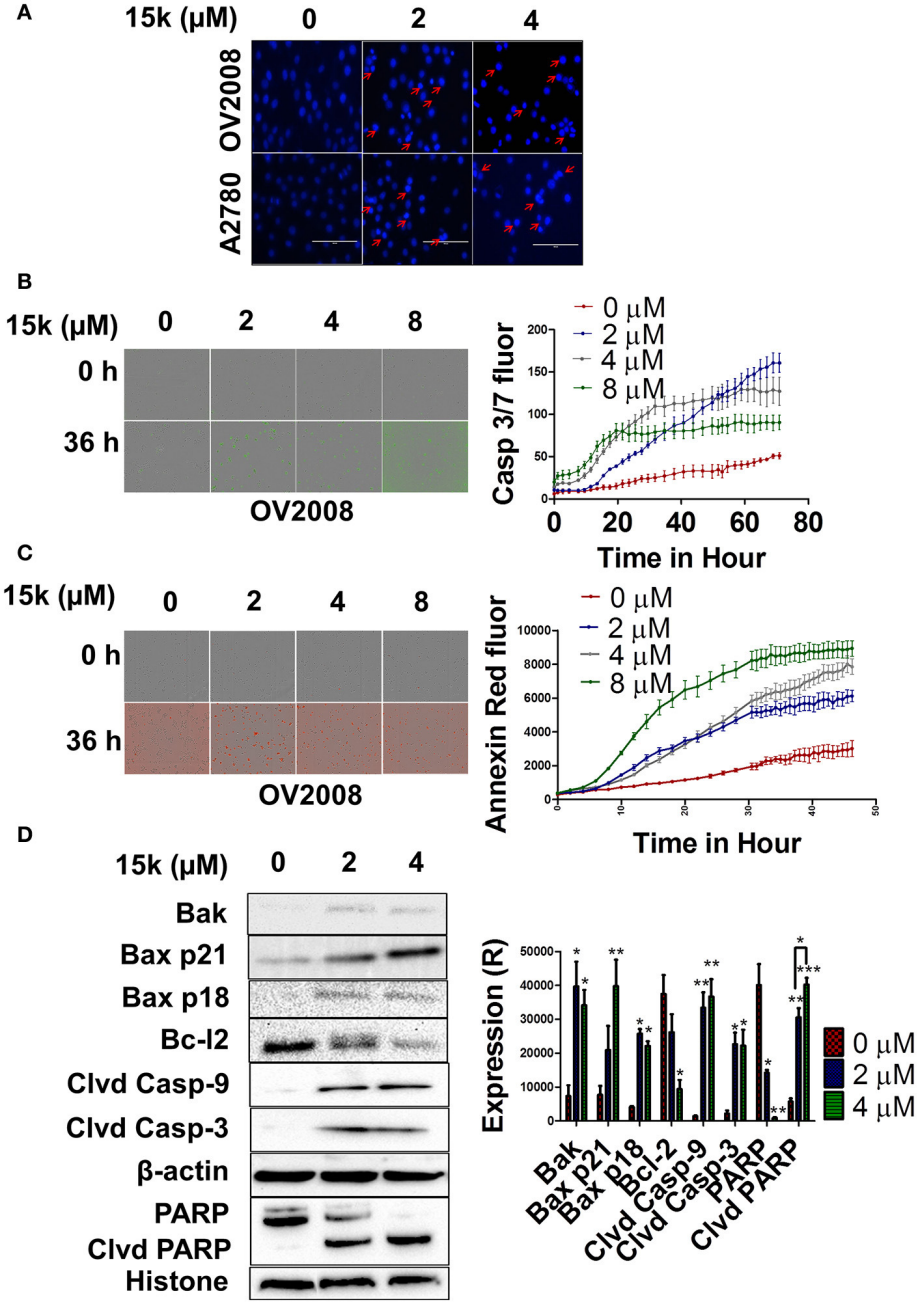
15k induces apoptosis through the intrinsic apoptotic pathway in OV2008 cells; **(A)** The nuclear fragmentation and apoptotic chromatin condensation (red arrows) due to **15k** (0, 2, and 4 μM) at 40x. **(B,C)** A real-time quantification of apoptosis after incubation of cells with **15k** (0, 2, 4, and 8 μM), using two IncuCyte fluorescent dyes (caspase 3/7 reagent and Annexin V red, respectively), in OV2008 cells. The caspase 3/7 reagent yields a green fluorescence and Annexin V produces red fluorescence. The pictures illustrate the fluorescence (fluor) level of the two reagents at the 0 and 36 h time points. In addition, the detailed results are shown in the time line curves next to the pictures. **(D)** The Western blot expression levels for the proteins Bak, Bax p21, Bax p18, Bcl-2 cleaved caspase-9, cleaved caspase-3, nuclear PARP and nuclear cleaved PARP following incubation with **15k** (0, 2, or 4 μM), where Clvd is for cleaved, Casp for caspase. β-actin was used as a cytosolic reference protein and histone as a reference for nuclear proteins. A histogram summarizing the result is shown next to the Western blots where (R) is for relative. The results are presented as the means ± SEM. ^*^p < 0.05, ^**^p < 0.01, ^***^p < 0.001 vs. the control group.

The incubation of OV2008 and A2780 cells with 2 or 4 μM of **15k** induced a significant nuclear condensation, with fragmented chromatin compared to vehicle-incubated cells (Figure 3A). In addition, the induction of apoptosis over time (real-time quantification) in OV2008 cells was detected using the two Incucyte reagents (caspase 3/7 and Annexin red). As seen in Figure 3B, OV2008 cells only had a relatively small increase in green fluorescence with time in the presence of vehicle, indicating that only a few cells underwent apoptosis after 3 days of incubation. In contrast, the incubation of cells with 2, 4, or 8 μM of **15k** induced apoptosis at 20, 15, and 15 h, respectively, as indicated by the significant increase in green fluorescence (Figure 3B). The increase in fluorescence induced by **15k** was maximal (i.e., apoptosis was maximal) after 72 h of incubation with all concentrations of **15k**. Similarly, cells incubated with vehicle had only a small increase in red fluorescence, indicating that there was a low level of apoptosis with time, even after 48 h of incubation (Figure 3C). However, cells incubated with **15k** had a significant increase in annexin red fluorescence over time (Figure 3C). Indeed, **15k** induced significant apoptosis after 24 h at 1 μM and ≈15 h at 4 and 8 μM (Figure 3C). The magnitude of apoptosis was the highest after 48 h of incubation with **15k**. Overall, our results indicate that **15k** significantly induces apoptosis at early time points.

Further experiments were conducted to determine the effect of **15k** on the level of the intrinsic apoptotic pathway proteins Bak, Bax, Bcl-2, and caspase 9. In addition, the expression levels of caspase 3 and its substrate, PARP, were also determined. The incubation of OV2008 cells with 2 or 4 μM of **15k** significantly increased the expression of Bak (*p* < 0.05 for both concentrations) compared to cells incubated with vehicle, where Bak expression was low (Figure 3D). Furthermore, 4 μM of **15k** significantly increased the levels of the proapoptotic protein, Bax (*p* < 0.001) (Figure 3D). Notably, **15k**, at 2 and 4 μM, also induced cleavage of the Bax p21 protein to a lower molecular weight fragment, Bax p18, in OV2008 cells (Figure 3D, *p* < 0.05 for both concentrations). In contrast, cells incubated with vehicle did not express significant levels of Bax p18 (Figure 3D). Interestingly, the cleaved p18 fragment is more efficacious than the p21 fragment in inducing apoptosis (Wood and Newcomb, 2000). Thus, our results suggest that the cleavage of Bax to p18 may be one of the primary mechanisms for **15k's** efficacy against OV2008. Compound **15k** (4 μM), compared to vehicle, also significantly inhibited the expression of the antiapoptotic protein, Bcl-2 (*p* < 0.05, Figure 3D). The cleaved active caspase 9 and caspase 3 were detected in OV2008 cells incubated with 2 or 4 μM of **15k**, whereas these caspases were not expressed in vehicle-treated cells (*p* < 0.01 for caspase 9 and *p* < 0.05 for caspase 3 for 2 and 4 μM, Figure 3D). Finally, the nuclear protein PARP, a substrate of caspase 3, was significantly cleaved in OV2008 cells incubated with **15k** compared to vehicle (Figure 3D). In contrast, cells incubated with vehicle had limited or no expression of cleaved PARP (Figure 3D). PARP expression was significantly reduced in a concentration - dependent manner by **15k** (*p* < 0.05 for 2 μM and *p* < 0.01 for 4 μM, Figure 3D. Collectively, our results indicate that **15k** induces apoptosis in OV2008 cell death by upregulating certain apoptotic proteins and downregulating key antiapoptotic proteins.

### 15k significantly inhibits the *in vitro* invasiveness and migration potential of OV2008 cells

**Figure 4:**
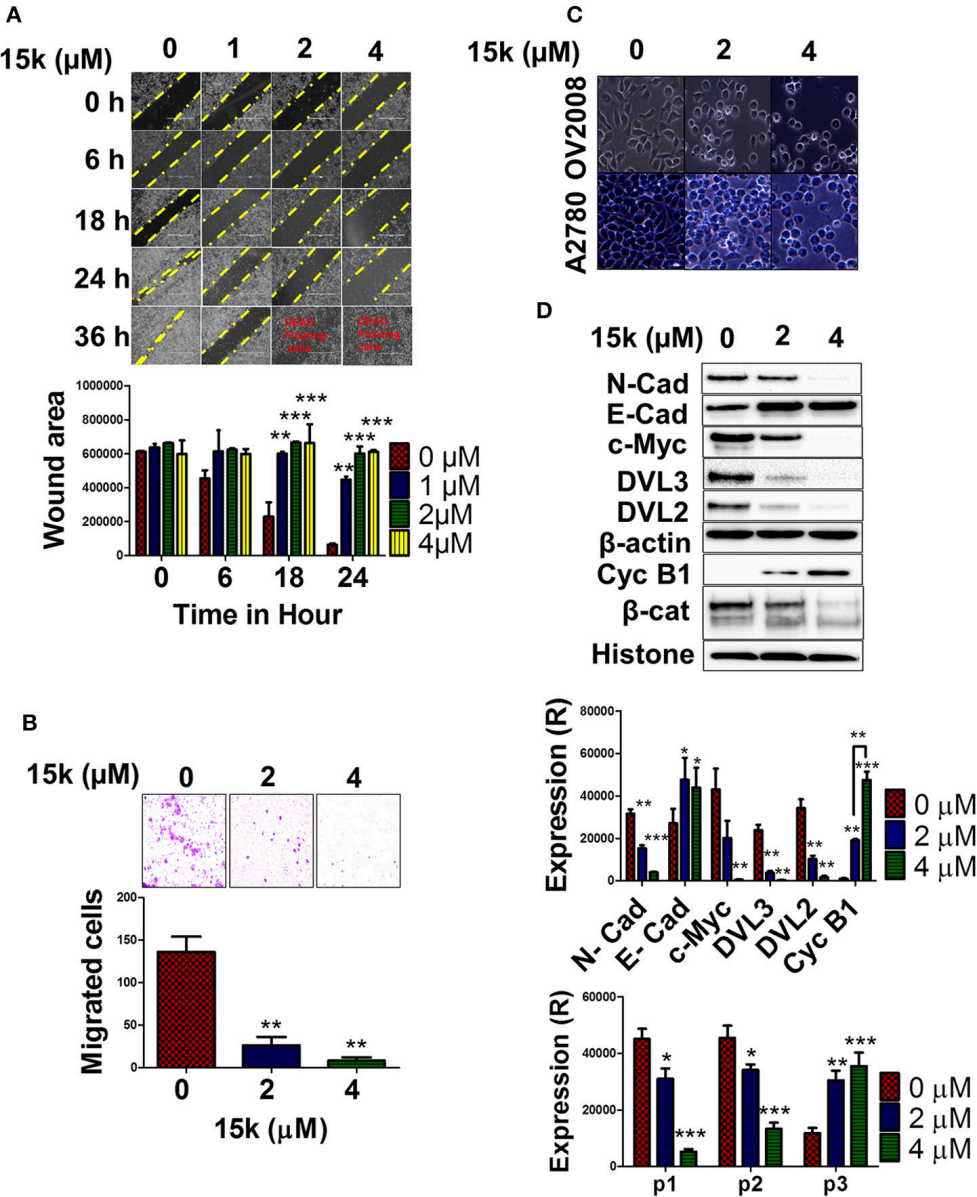
The *in vitro* effect of **15k** on OV2008 migration and invasiveness, as well as EMT/ Wnt/β-catenin proteins; **(A)** Representative images showing the wound area and closure at different time points (0, 6, 18, 24, and 36 h) after incubation with **15k** (0, 1, 2, and 4 μM). Below the images is a histogram quantitatively showing the results and statistical analysis of the data; **(B)** The transwell migration assay is shown as representative images of the level of cells that have migrated after incubation with **15k** (0, 2, and 4 μM). Below the images is a histogram showing the number of cells that have migrated and invaded the membrane inserts (counted/field of image); **(C)** A representative image of OV2008 and A2780 cell morphology taken from an EVOS microscope (40X) following incubation with **15k** (0, 2, and 4 μM). **15k** induced cells to transform from a spindle—mesenchymal shape, to a more round, epithelial shape; **(D)** Western blots for the expression of N-cadherin (N-cad), E-cadherin (E-cad), c-Myc, DVL3, DVL2, Cyclin B1 (Cyc B1) and β-catenin (β-cat) fragmentation. Cells were incubated with (0, 2, and 4 μM) of **15k**. β-actin levels were used to normalize cytosolic proteins and histone was used for normalizing nuclear proteins. Below the Western blots is a histogram quantitatively summarizing the results where (R) is for relative. The data are presented as the means ± SEM of three independent experiments. ^*^p < 0.05, ^**^p < 0.01, ^***^p < 0.001 vs. control group.

In order to determine if **15k** has antimetastatic efficacy in OV2008 cells, two well-established, *in vitro* cell-based assays for migration and invasiveness were used, the wound healing and matrigel transwell migration assays, respectively (Justus et al., 2014). In the wound-healing assay, OV2008 cells incubated with **15k** were significantly less mobile and migratory compared to control cells (Figure 4A). The control cells migrated relatively quickly, resulting in complete wound closure 36 h after the wound was created (Figure 4A) However, wound closure was significantly decreased in OV2008 cells incubated with 1 (*p* < 0.01 at 18 and 24 h), 2 (*p* < 0.001 at 18 and 24 h) or 4 (*p* < 0.001 at 18 and 24 h) μM of **15k** (Figure 4A). Furthermore, the majority of the OV2008 cells were non-viable after 36 h of incubation with **15k** (Figure 4A). Additionally, the incubation of OV2008 cells with 2 or 4 μM (*p* < 0.01 and <0.001, respectively) significantly decreased their migration across a semipermeable membrane compared to vehicle-incubated cells (Figure 4B). Thus, *in vitro*, **15k** significantly inhibits the migration and invasive potential of OV2008 cells.

### 15k significantly inhibits EMT signaling in OV2008 cells

We conducted experiments to determine the mechanism by which **15k** decreases the migration and invasiveness of OV2008 cells. Previous studies suggest that the parent compound of **15k**, silybin, reverses ovarian cancer metastasis by attenuating signaling in the EMT and Wnt/β-catenin pathways (Ting et al., 2013; Eo et al., 2016). Therefore, we determined the effect of **15k** on the signaling, as well as cell morphology, in the aforementioned pathways. As shown in Figure 4C, OV2008 cells incubated with vehicle have an elongated, spindle-like morphology, indicative of the maintenance of the mesenchymal phenotype. In contrast, OV2008 cells incubated with 2 or 4 μM of **15k** had a significant change in their morphology, characterized by a rounded, epithelial-like phenotype (Figure 4C). Similar morphological changes were seen in A2780 cells following incubation with **15k**.

Subsequently, we determined the effect of **15k** on the expression level of the following proteins in the EMT and Wnt/β-catenin pathways: N-cadherin, E-cadherin, c-Myc, DVL3, DVL2, cyclin B1 and β-catenin. The incubation of OV2008 cells with 2 or 4 μM of **15k** significantly decreased (*p* < 0.01 for 2 μM and *p* < 0.001 for 4 μM) the expression of the mesenchymal marker, N-cadherin (Figure 4D). However, 2 and 4 μM of **15k** significantly increased (*p* < 0.05 for both concentrations) the levels of the epithelial marker, E-cadherin, compared to cells incubated with vehicle (Figure 4D).

The incubation of OV2008 cells with 4 μM of **15k** significantly decreased (*p* < 0.001) the levels c-Myc, a protein that induces EMT signaling (Cho et al., 2010), compared to cells incubated with vehicle Figure 4D). The incubation of OV2008 cells with 2 or 4 μM of **15k** significantly increased (*p* < 0.01 for 2 μM and *p* < 0.001 for 4 μM) the levels of cyclin B1 (Figure 4D) compared to cells incubated with vehicle. Moreover, 4 μM of **15k** produced a significantly greater (*p* < 0.01) increase in cyclin B1 levels compared to 2 μM (Figure 4D).

We also determined the effect of **15k** on 3 key regulatory proteins in the Wnt/β-catenin pathway: DVL3, DVL2, and β-catenin. The incubation of OV2008 cells with 2 or 4 μM of **15k** significantly decreased the levels of both DVL3 and DVL2 (*p* < 0.01 for both proteins and concentrations of **15k**) compared to cells incubated with vehicle (Figure 4D). Interestingly, **15k** induced the fragmentation (a novel finding) in the β-catenin protein compared to cells incubated with vehicle (Figure 4D). As can be seen in Figure 4D, vehicle-incubated OV2008 cells expressed two high molecular weight fragments primarily p1 or p92 and to a lesser extent, p2 or p85. However, 2 and 4 μM of **15k** significantly decreased the expression of p 92 (*p* < 0.05 for 2 μM and <0.001 for 4 μM, Figure 4D). Furthermore, cells incubated with 2 or 4 μM of **15k** expressed a novel, lower molecular weight fragment, p3, with a lower molecular weight, that was not expressed in OV2008 cells incubated with vehicle (*p* < 0.01 for 2 μM and *p* < 0.001 for 4 μM, Figure 4D). Thus, the **15k** induced fragmentation of β-catenin is primarily activated by apoptosis, enhancing its antiproliferative and antimetastatic efficacy in OV2008 cells.

**Figure 5:**
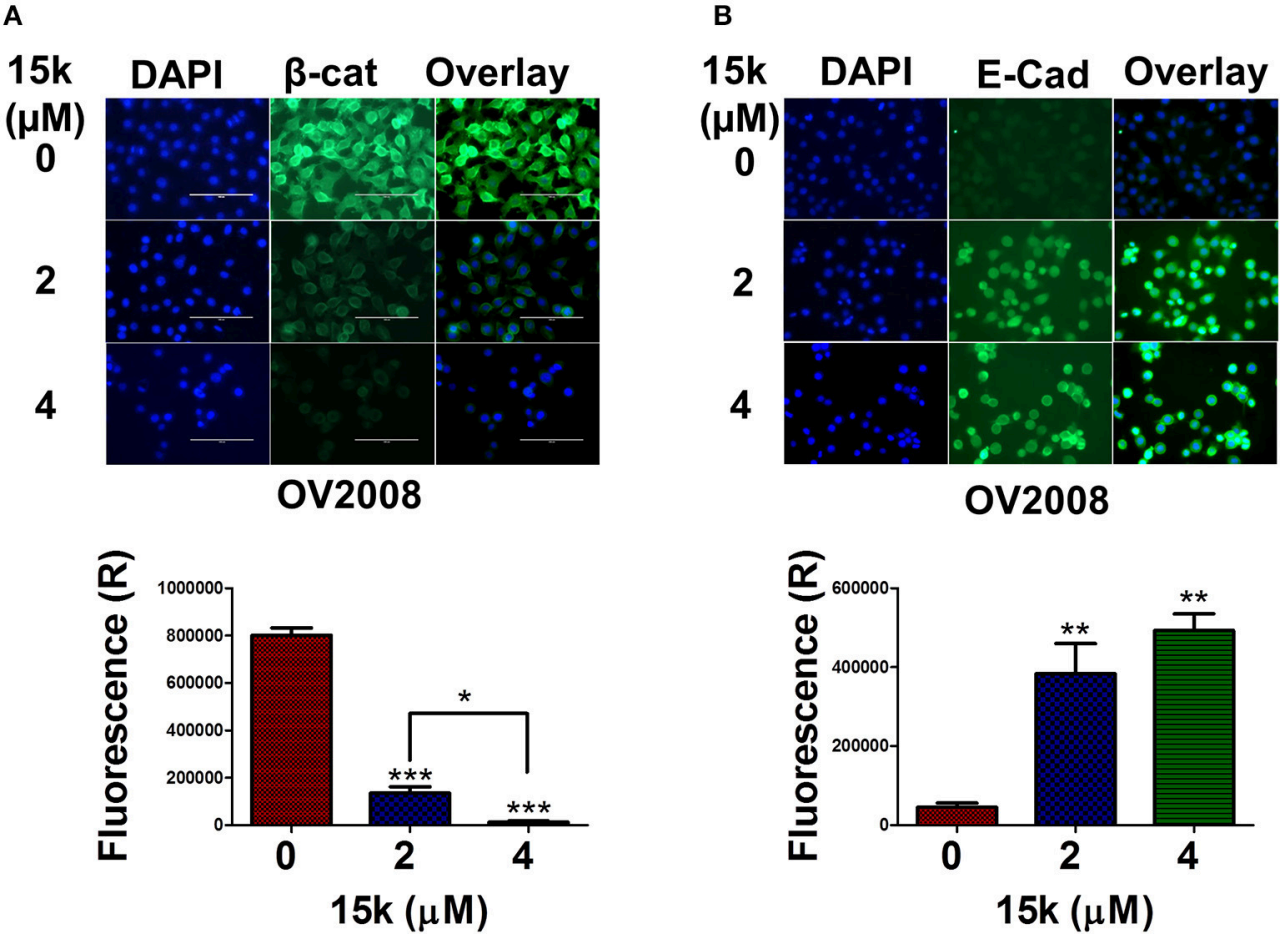
The effect of **15k** on the expression levels of β-catenin (β-cat) and E-cadherin (E-Cad) in OV2008 cells using immunofluorescent staining. Cells were incubated with **15k** (0, 2, and 4 μM). **(A)** Representative pictures of β-catenin fluorescence in OV2008 cells. **(B)** Representative pictures of E-cadherin fluorescence in OV2008 cells. Below each protein picture is a histogram summarizing the results where expression (R) is the relative expression. The data are presented as the means ± SEM of three independent experiments. ^*^p < 0.05, ^**^p < 0.01, ^***^p < 0.001.

Immunofluorescent staining was used to determine the effect of **15k** on the levels of β-catenin and E-cadherin (Figure 5). OV2008 cells incubated with vehicle had a high level of green fluorescence, indicating high levels of the β-catenin protein. However, the incubation of OV2008 cells with 2 or 4 μM of **15k** significantly decreased (*p* < 0.001) green fluorescence (Figure 5A), with the decrease being greater at 4 vs. 2 μM of **15k**. In Figure 5A, it can be seen that the nuclear translocation of β-catenin was reduced as fluorescence is limited to the cytosol, with no fluorescence in the nuclear area (Figure 5A). The expression levels were also significantly lower with higher concentration (4 μM) compared to lower concentration (2 μM) with *p* < 0.05, Figure 5A).

In contrast to β-catenin expression, the fluorescence levels of E-cadherin were significantly upregulated in OV2008 cells incubated with 2 (*p* < 0.01) or 4 μM (*p* < 0.01) of **15k** compared to vehicle (Figure 5B). The control OV2008 cells had a very low fluorescence, indicating the predominance of the mesenchymal phenotype. The above results suggest that **15k** reverses the mesenchymal and migratory phenotype in OV2008 cells allowing the expression of the epithelial, non-invasive and non-migratory phenotype.

### 15k significantly downregulates cell lines overexpressing ABCG2 and ABCB1 transporters

**Figure 6:**
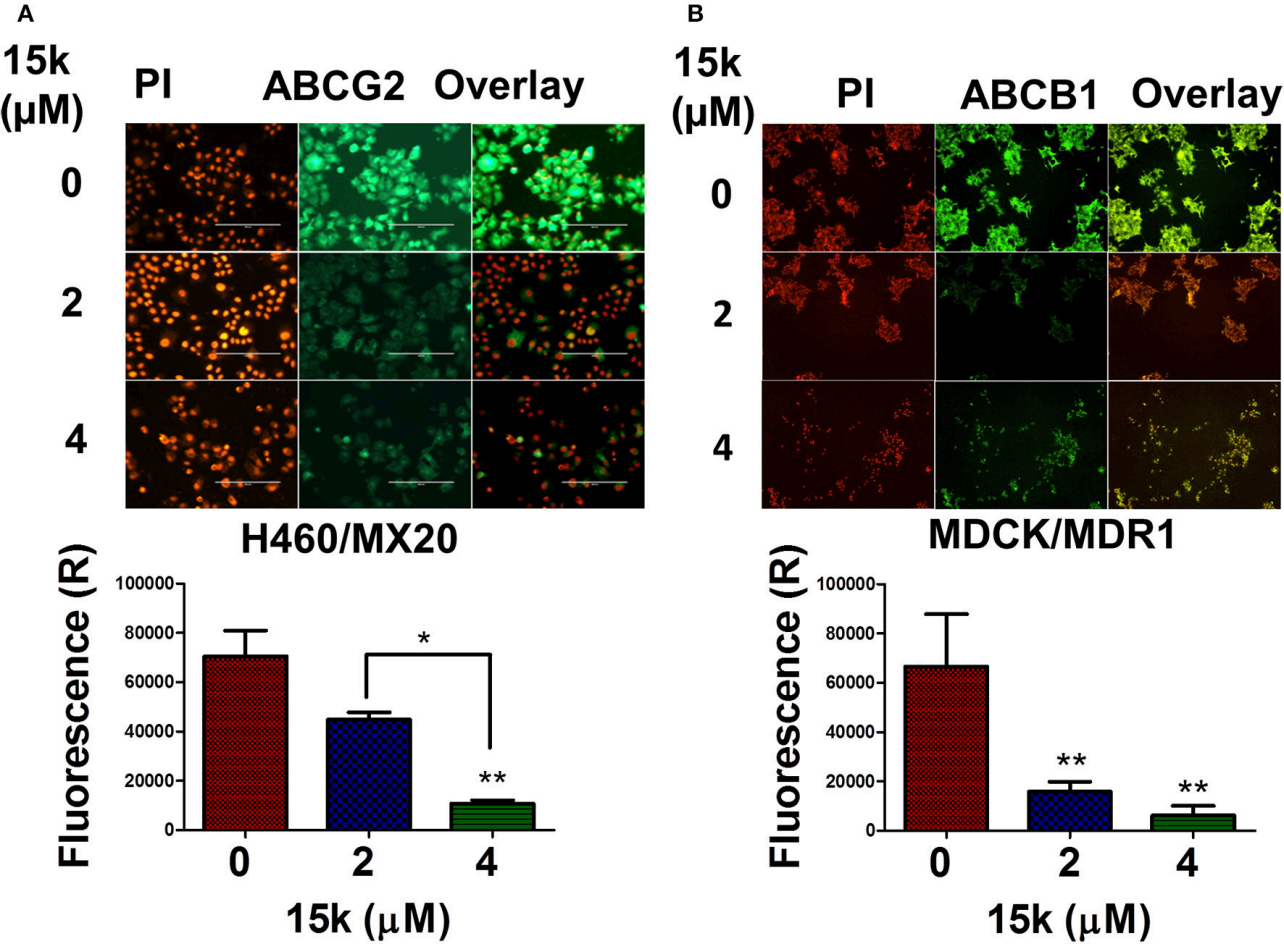
The effect of **15k** on the expression level of ABCG2 transporters in H460/MX20 cells and ABCB1 transporters in MDCK/MDR1 cells. **(A)** Representative pictures of ABCG2 fluorescence in H460/MX20 cells. **(B)** Representative pictures of ABCB1 fluorescence in MDCK/MDR1 cells. Below each of the protein pictures is a histogram summarizing the results where (R) is for relative. The data are presented as the means ± SEM of three independent experiments. ^*^p < 0.05, ^**^p < 0.01.

One of the major mechanisms by which cancer cells become resistant to numerous structurally and functionally unrelated anticancer drugs is the overexpression of specific ABC transporters (Choi, 2005). There are also data indicating that EMT-inducing transcription factors can increase the expression of ABCG2 and ABCB1 transporters (Saxena et al., 2011; Du and Shim, 2016). Therefore, we determined the effect of **15k** on the expression of ABCG2 transporters in H460/MX20 and ABCB1 transporters in MDCK/MDR1 cells, which overexpress ABCG2 and ABCB1 transporters, respectively, using an immunofluorescent staining assay (Figure 6). The incubation of H460/MX20 cells with 4 μM, but not 2 μM, of **15k** significantly reduced (*p* < 0.01) green fluorescence compared to cells incubated with vehicle (Figure 6A). The **15k**-induced reduction in the fluorescence is indicative of the downregulation of ABCG2 transporter expression. Similarly, a significant decrease in the expression of fluorescence in MDCK/MDR1 cells was produced by 2 or 4 μM of **15k** (*p* < 0.01 for both concentrations, Figure 6B). Although we determined the effect of **15k** on the expression of these transporters in non-ovarian cell lines, the inhibition of ABCG2 and ABCB1 transporters in these cells suggests that **15k** may reverse multidrug resistance in ABCB1 and ABCG2 overexpressing cells lines.

### 15k safety in zebrafish

**Figure 7:**
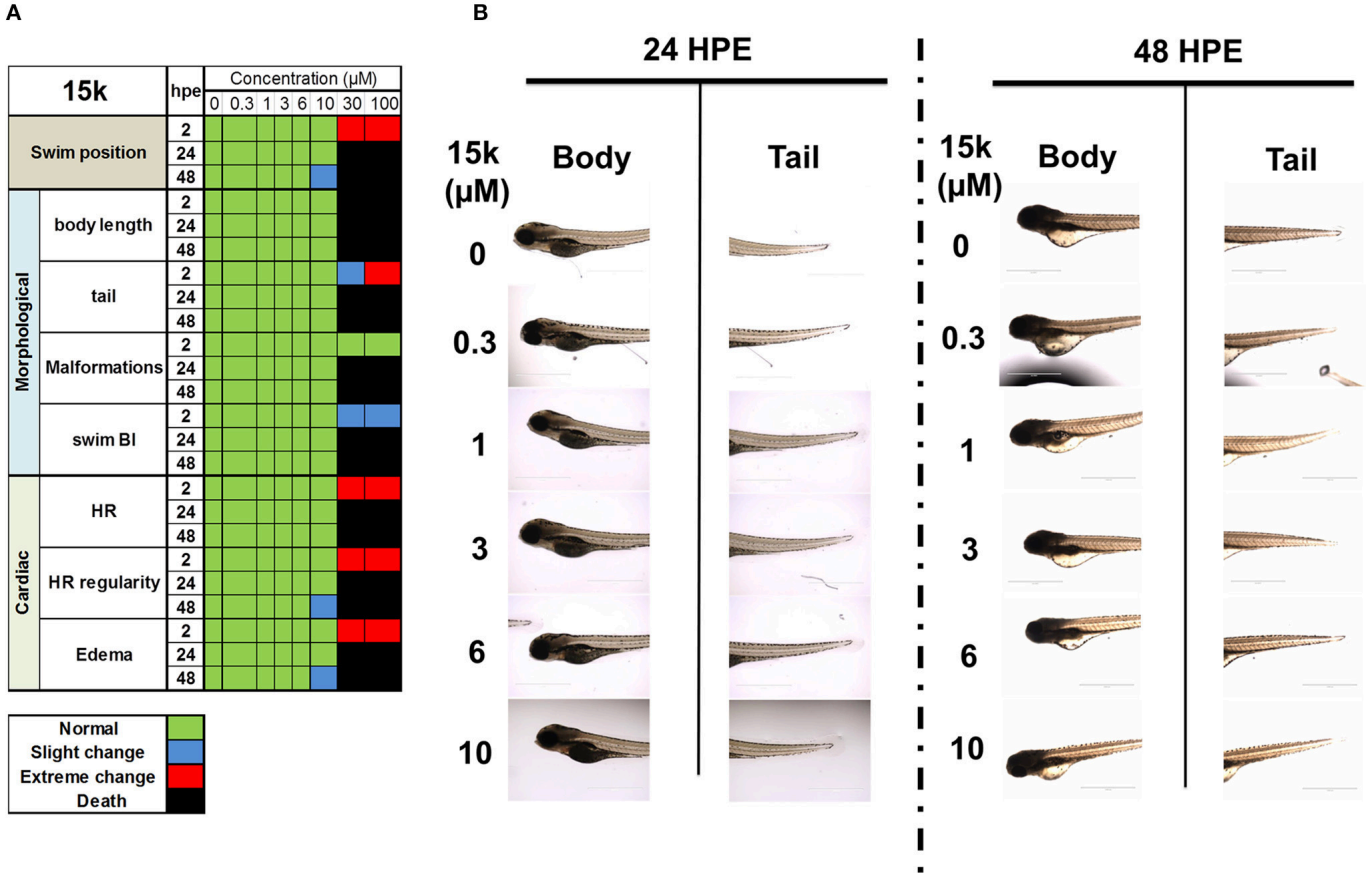
The safety of **15k** in zebrafish *in vivo* model. **(A)** A heat map table showing the effect of **15k** on the swimming position, morphological, and cardiac parameters of the fish at 0, 0.3, 1, 3, 6, 10, 30, and 100 μM for 2, 24, and 48 hpe; **(B)** The shape of the body and tail of zebrafish after treatment of **15k** at 0.3, 1, 3, 6, 10, 30, and 100 μM for 24 and 48 hpe.

To further verify the safety of **15k** *in vivo*, the zebrafish model was used. The results of the changes in cardiac, morphology and swimming position parameters are shown in the heat map in Figure 7A. **15k** did not produce significant toxicity in zebrafish at concentrations up to 10 μM. The different groups showed similar body lengths, body shapes, tails, swim bladders and fins (Figures 7A,B). There were no significant differences in the mortality rate in any of the groups exposed to 15k compared to the control group at 2, 24, and 48 hpe (Figure 7A). At 10 μM, most of the fish were healthy and alive. However, at 48 h, a few of the fish developed minor changes in HR and a slight loss of dorsoventral balance. Significant malformations, like yolk retention, swim bladder absence and tail changes, were not detected (see the shapes of the zebrafish, Figure 7B). However, at 30 and 100 μM, the animals developed severe toxicities, such as curved body shape, severe fluids retention, pericardial edema and severe HR irregularities at 2 hpe and there was a significant increase in mortality at 24 and 48 hpe (100% death, Figure 7A). Overall, our results indicate that the acute toxic concentration of **15k** is higher than 10 μM and zebrafish can tolerate up to 10 μM of **15k**.

## Discussion

In this study, we conducted experiments to determine the mechanism of action of our novel sylibin derivative, **15k**, using OV2008 cells. One of the major findings was that **15k**, at 1, 2, 4 and 8 μM significantly inhibited OV2008 proliferation (IC_50_ < 1 μM) and colony formation rate. Compared to silybin, **15k** inhibits ovarian cancer cell proliferation at significantly lower concentrations. Previously, silybin's antiproliferative efficacy in ovarian cancer was studied both *in vitro* and *in vivo* (Gallo et al., 2003; Cho et al., 2013). Silybin inhibited the growth of ovarian cancer cells (A2780 and SKOV3) in a time- and concentration - dependent manner (Cho et al., 2013). However, silybin only produced antiproliferative efficacy at high concentrations. For example, 58−65% of the cancer cells were viable after 48 h of incubation with 50 μM of silybin, compared to 98% of normal ovarian cells (OSE) (Cho et al., 2013). It has been reported that only 150 and 200 mg/ml of silymarin resulted in 60–70 % cell death (30–40% survival) after 24 h of incubation in the ovarian cancer cell lines A2780 and SKOV3 (Fan et al., 2014). Furthermore, silybin, at concentrations from 1 to 10 μM, did not significantly decrease the viability of A2780 cells (cell survival > 90%) (Giacomelli et al., 2002). We previously reported that **15k** had significant cytotoxic efficacy (IC_50_ ≈1 μM) in ovarian cancer cells (OV2008 and A2780) that was 30–200-fold greater than silybin (IC_50_ ~30–200 μM on OV2008 and A2780 cells). Additionally, **15k**, like silybin, was significantly more cytotoxic in ovarian cancer cells compared to the normal cell lines, Chinese hamster ovarian cells (CHO, IC_50_ = 8.1 ± 1 μM) and normal colon epithelial cells (CRL-1459, IC_50_ = 8.5 ± 0.7 μM, ≈ 10x selectivity) (Manivannan et al., 2017).

**15k** significantly induced the generation of ROS at 1, 2, and 4 μM after 24 and 48 h of incubation in OV2008 cells. The generation of high levels of ROS has been reported to result in extensive cell damage, apoptosis and death in cancer cells (Circu and Aw, 2010). ROS, such as hydrogen peroxide, can cause cell damage by altering cellular macromolecules, such as DNA, proteins, and lipids (Andreyev et al., 2005). Previously, it has been suggested that certain anticancer drugs may produce their efficacy, in part, by generating high levels of ROS (Conklin, 2004). Similarly, our results suggest that **15k** also induced the formation of ROS in OV2008 cells, which could contribute to a loss of cell viability.

Apoptosis is characterized by cell shrinkage, significant chromatin condensation, and phosphatidyl serine (PS) relocalization to the external side of cell membrane (Elmore, 2007; Tan et al., 2009). Our results indicated that **15k** significantly induced apoptosis in a concentration- and time - dependent manner that was detected by DAPI nuclear staining (2 and 4 μM) and the Incucyte annexin red and caspase 3/7 assays (2, 4, and 8 μM). Indeed, **15k** elicited nuclear condensation and chromatin fragmentation, as well as an increase in annexin red (binds to PS) and caspase 3/7 levels, as indicated by the increases in green and red fluorescence, respectively. Previously, several studies reported that silybin induces cancer cell apoptosis (Agarwal et al., 2003; Fan et al., 2014; Wang et al., 2014). For example, 50 μM of silybin increased apoptosis from 0.25% in the controls to 42.09% and from 0.21 to 26.19% in A2780 and SKOV3 cells, respectively (Cho et al., 2013). Another study reported that only 100 μM of silybin induced significant apoptosis in in A2780 ovarian cells (Fan et al., 2014). The combination of silybin 100 μM with paclitaxel induced higher level of apoptosis in A2780 cells resistant to taxol (Zhou et al., 2008). Compared to silybin, **15k** induced significant apoptotic cell death in two ovarian cancer cell lines, OV2008 and A2780, at significantly lower concentrations (2, 4, and 8 μM for **15k** vs. 100 μM for silybin). Our results indicated that **15k** promoted nuclear condensation and increased annexin red and caspase 3/7-reagent fluorescence at relatively early time points (<24 h).

A novel finding of this study was that **15k** induced the cleavage of Bax p21 into Bax p18. Thus, it is likely that one of the primary mechanisms of action of **15k** is due to its induction of the cleavage of Bax p21, producing cell death and apoptosis in OV2008 cells. The cleavage of Bax p21 to the p18 fragment by various compounds has been previously reported (Thomas et al., 1996; Wood et al., 1998). For example, in B-cell chronic lymphocytic leukemia (B-CLL), the camptothecin analog, 9-amino-20(s)-camptothecin, and the purine analog, fludarabine, resulted in the cleavage of Bax p21 to p18, producing a greater magnitude of apoptosis (Thomas et al., 1996). The cleavage of Bax p21 in HL-60 cells also occurs after incubation with the 4 μM of the topoisomerase I inhibitor, 9-AC (9-aminocamptothecin) (Wood et al., 1998). Furthermore, the caspase inhibitor, carbobenzoxy-valyl-alanyl-aspartyl-(O-methyl)-fluoromethylketone (z-VAD-fmk), can completely block apoptosis induced by p21 Bax in HEK 293T cells, but only partially block apoptosis induced by p18 Bax (Wood and Newcomb, 2000). The incubation of Jurkat T cells with 50 μM of etoposide or 1 μM of staurosporin resulted in the cleavage of Bax p21 to Bax p18, cytochrome c release, caspase 3 activation, cleavage of PARP and DNA fragmentation (Gao and Dou, 2001). The apoptosis induced by Bax p18 occurred in the presence of high levels of Bcl-2 (an antiapoptotic protein) (Gao and Dou, 2001). To the best of our knowledge, silybin has not been reported to induce Bax cleavage in any cancer cell line. Notably, our results indicate that **15k**, in contrast to its parent compound silybin, induces apoptosis via Bax p21 cleavage. Our results also indicated that **15k** decreased the expression of Bcl-2, activated caspases 3 and 9, and induced the cleavage of PARP and β-catenin. Bcl-2 is located at the mitochondrial membrane and it functions to prevent the release of important apoptogenic factors to the cytoplasm, including cytochrome c (Tsujimoto, 1998). The Bax/Bcl-2 ratio is one index that determines the level of apoptosis induction and cell fate as downregulation of Bcl-2 significantly increases apoptosis (Bagci et al., 2006). The compound **15k**, at 2 and 4 μM, significantly increased the Bax/Bcl-2 to ratio by increasing Bax and decreasing Bcl-2 expression. Furthermore, **15k** significantly increased the activation of caspases 3 and 9, leading to the activation of the caspase cascade that results in the cleavage of key cellular proteins, producing cell death (Adams and Cory, 1998). Previously, it has been reported that he incubation of A2780 cells with 50 or 100 μg/ml of silymarin for 24 h resulted in significant downregulation in intact procaspase 3 and procaspase 9, with significant upregulation in the levels of the active cleaved forms, caspase 3 and caspase 9 (Fan et al., 2014). Thus, **15k** (2 and 4 μM) was significantly more potent than silybin in inducing the activation of caspase 9 and caspase 3 in OV2008 cells, thereby increasing the likelihood of apoptosis.

In this study, compound **15k** (2 and 4 μM) significantly induced the cleavage of the protein PARP. PARP has been shown to repair damaged DNA and regulate chromatin structure (Morales et al., 2014). Furthermore, the cleavage of PARP by caspase 3 is one of the major events that occur during the last stages of apoptosis (Nicholson et al., 1995; Le Rhun et al., 1998; Boulares et al., 1999). Previous studies examining the effect of silybin analogs have been reported. The incubation of LNCap and 22Rv1 cells with isosilybin A and B (60 and 90 μM, respectively) for 48 h induced the cleavage of PARP (Deep et al., 2007). In Hela cells, 2,3-dehydrosilybin, at 25 and 50 μM, activated the cleavage of caspase 3, caspase 9 and significantly induced PARP cleavage. This is in contrast to the results obtained with our silybin derivative, **15k**, which induced the cleavage of both nuclear and cytosolic PARP at 2 and 4 μM. Overall, **15k** is inducing apoptosis in OV2008 cells at relatively low concentrations through the upregulation of Bax, Bax cleavage, downregulation of Bcl-2, activation of caspases-3, caspase-9 and PARP cleavage.

The incubation of OV2008 cells with **15k** (1, 2, and 4 μM) significantly decreased their metastatic potential as it inhibited cell invasiveness and migration *in vitro*. Previously, it has been reported that the anticancer efficacy of silybin was not limited to primary tumors (Deep and Agarwal, 2010). Silybin, *in vitro*, (50–200 μM) also significantly decreases metastasis and the invasiveness of certain types of cancer (Wood et al., 1998; Deep and Agarwal, 2010). Data from several *in vitro* models indicate that silybin was efficacious in reversing wound healing and transwell migration of different cancer cells (Wu et al., 2009; Deep et al., 2014). In A2780 ovarian cancer cells, silybin (100 μM) inhibited metastasis and invasiveness by >50 % inhibition in the resistant ovarian cancer cell line, A2780/taxol (Zhou et al., 2008). In this study, **15k** at significantly lower concentrations than silybin or silibinin, reversed OV2008 migration and invasiveness *in vitro* based on the results of the wound healing and transwell migration assays. Tentatively, these results suggest **15k** may have efficacy against metastatic ovarian cancer cells. Further studies must be conducted to determine if **15k** produces antimetastatic efficacy in an *in vivo* model.

The overactivation of the epithelial - mesenchymal transition (EMT) pathway has been reported to induce cancer growth, progression, and metastasis (Deep et al., 2011; Heerboth et al., 2015). When cells undergo EMT, the immobile adherent cells become invasive, mobile cells and have the potential to migrate (Heerboth et al., 2015). The transition of cells from the epithelial to mesenchymal phenotype is associated with the loss of E-cadherin protein, whereas N-cadherin levels are increased (Huber et al., 2005). The E- cadherin is tumor suppression gene that regulates epithelial cell behavior (van Roy and Berx, 2008). The loss of E-cadherin results in the loss of cell adhesion and initiation of invasive, mesenchymal phenotypes (Becker et al., 1994; Efstathiou et al., 1999). In contrast, N-cadherin promotes tumor invasion and produces significant morphological changes in epithelial cells, making them more motile and invasive (Derycke and Bracke, 2004). We hypothesized that the inhibition of OV2008 cell migration and invasiveness by **15k** is due to its effect on certain proteins in the EMT/beta-catenin pathway. Indeed, our data indicated that **15k** (2 and 4 μM) significantly upregulated the expression of the protein E-cadherin and down regulated the N-cadherin. Previously, silybin has been reported to inhibit EMT in various types of cancer cells (Cufi et al., 2013). The incubation of the migratory prostate cancer cell lines, PC3 and PC3MM2, with different concentrations of silybin (up to 90 μM) significantly 1) increased the expression of E-cadherin and 2) decreased nuclear β- catenin levels (Deep et al., 2014). Silybin, at 100 μM, inhibited the invasiveness of the metastatic prostate cancer cell lines, ARCaPM and DU145 and decreased the expression of mesenchymal biomarkers in these cell lines. In ovarian cancer cells (A2780), both *in vitro* and *in vivo*, sylibin (50 μM) significantly inhibited the phosphorylation of both Akt and Erk proteins, which induce EMT. Our results indicated that the silybin derivative **15k**, at 2 and 4 μM, concentrations lower than those reported for silybin, significantly reversed the mesenchymal phenotype in OV2008 ovarian cancer cells to the epithelial, and rounded phenotype. Notably, **15k** inhibited N-cadherin expression and upregulated E-cadherin levels. Thus, *in vitro*, **15k** produces significant antiproliferative and antimetastatic efficacy in OV2008 ovarian cancer cells by affecting the levels of key proteins involved in the EMT of ovarian cancer cells.

The Wnt/β-catenin pathway has been shown to be involved in the metastasis of cancer (Basu et al., 2016). β-catenin, a key regulator protein, is translocated to the nucleus after activation, where it binds to the T-cell factor/lymphoid enhancer factor (TCF/LEF) complex and induces the expression of several oncogenes, including c-Myc, which promote cancer and metastasis (Polakis, 2012). The disheveled proteins, DVL2 and DVL3, stabilize β-catenin and prevent its degradation by the enzyme glycogen synthase kinase 3 beta (GSK3β) (MacDonald et al., 2009) Both the EMT and Wnt/β-catenin pathways are highly connected and can activate each other through a cycle of positive feedback mechanisms (Wu et al., 2012). Although there are data indicating that the loss of E-cadherin can increase the cytoplasmic and nuclear levels of β- catenin (Eger et al., 2000), there are reports indicating that β-catenin interaction with the LEF1 transcriptional receptor in the nucleus forms a cooperative complex that result in zinc finger transcription factor (snail) - mediated repression of the E-cadherin expression by affecting its promotor region (Jamora et al., 2003; Yook et al., 2005). Furthermore, the loss of Wnt/β-catenin signaling significantly inhibits EMT, resulting in the upregulation of E-cadherin (Bernaudo et al., 2016). Our results clearly indicate that 15k cleavesβ-catenin, resulting in the upregulation of E-cadherin. Therefore, the upregulation of E-cadherin is probably the result of β-catenin cleavage and not *vice versa*.

The c-Myc oncogene, when transcribed, produces the protein c-Myc, an activator of Wnt/B-catenin and EMT (Cho et al., 2010). The upregulation of c-Myc is associated with E-cadherin suppression, N-cadherin upregulation, and β-catenin stabilization (Cowling and Cole, 2007). c-Myc is also upregulated by the nuclear translocation of β-catenin (Adams and Cory, 1998). Silybin has been reported to affect the Wnt/β-catenin pathway in several cancer cell lines (Vaid et al., 2011; Eo et al., 2016). Silybin, at 50 and 100 μM, has been shown to inhibt β-catenin expression and its transcriptional functions in HCT116, SW480, A375, and Hs294t (Vaid et al., 2011; Eo et al., 2016). Similarly, silybin also inhibited c-Myc expression in HCT116 colon cancer cells at 50 and 100 μM (Eo et al., 2016). However, to date, there are no published reports on the effect of silybin on Wnt/β-catenin signaling in ovarian cancer cells. Our results, in OV2008 ovarian cancer cells, indicate that **15k**, at 2 and 4 μM, significantly decreased the levels of DVL3, DVL2 and c-Myc.

The cleavage of β-catenin has only been reported by a few studies. For example, 60 μM of resveratrol, added to Hela cells for 48 h, induced both caspase-3 and proteasomal cleavage of β-catenin (Ray et al., 2015). However, there are no published data indicating that silybin induces the cleavage of β-catenin in cancer cells. The cleavage of β-catenin during apoptosis is caspase 3 – dependent, resulting in the generation of several lower molecular fragments (Steinhusen et al., 2000). Functional analysis has shown that these lower molecular weight fragments have lower transcriptional functionality compared to un-cleaved, full length β-catenin (Steinhusen et al., 2000). Our silybin derivative, **15k**, at 2 and 4 μM, induced significant activation of caspase 3 - dependent apoptosis in OV2008. Furthermore, **15k** induced significant cleavage of β-catenin to lower molecular weight fragments. Therefore, one of the novel mechanisms of action of **15k** is its caspase 3 - dependent cleavage of β-catenin.

There are data indicating that the EMT/Wnt/β-catenin pathways are dysregulated or overactivated in ovarian cancer cells (Vergara et al., 2010). Therefore, inhibiting multi-targets in these two pathways could represent an anticancer strategy against both primary and metastatic ovarian cancer. Compound **15k**, at relatively low concentrations, significantly affects key regulatory proteins in the EMT/Wnt/β-catenin pathways. These data, in combination with the phase-contrast morphological changes support our conclusion that **15k** acts by inhibiting the EMT pathway. In addition, these aforementioned results were augmented by our immunofluorescence data indicating that **15k** induced a significant upregulation in E-cadherin levels and further inhibited the translocation of β-catenin from the cytosol to nucleus. Nevertheless, there are several EMT factors, such as vimentin, snail, twist, zeb, and cytokeratin, among others, that are important. Consequently, future studies should be conducted to determine the effect of 15k on these proteins to further understand the mechanism of **15k** on the EMT pathway.

Multidrug resistance (MDR) is a major challenge in cancer treatment, contributing significantly to treatment failure and disease recurrence (Chang, 2003). It is well established that the overexpression of p-glycoprotein (P-gp or ABCB1) (Krech et al., 2012) and breast cancer resistance protein (BCRP or ABCG2) (Natarajan et al., 2012) play a major role in mediating MDR in certain types of cancer cells (Krech et al., 2012; Natarajan et al., 2012). Silybin analogs have been reported to interact with ABC transporters. For example, dehydrosilybin binds with high affinity to the cytosolic domain of p-glycoprotein-like transporters, inducing the sensitization of multidrug-resistant Leishmania spp. to daunomycin (Perez-Victoria et al., 2001). Silymarin (50–150 μM) inhibits ABCB1-mediated drug efflux in Caco2 cells and increased the accumulation of ^3^H - digoxin (50–150 μM) and ^3^H-vinblastine (150 μM) (Abdallah et al., 2015). The combination of silybin (100 μM) with paclitaxel (100 nM) significantly reduced resistance (from 5.2 to 1.5-fold) to taxol in A2780 ovarian cells (Zhou et al., 2008). Silybin (100 μM) also significantly inhibited ABCB1 transporters in taxol - resistant A2780 cells (Zhou et al., 2008). Thus, based on the above findings, we determined the effect of our silybin derivative, **15k**, on ABCB1 and ABCG2 transporters in cell lines that overexpressed these transporters. Compound **15k** (2–4 μM) significantly downregulated (1) ABCG2 expression in resistant lung cancer cells (H460/MX20) and (2) ABCB1 expression in resistant madin-darby canine kidney cells (MDCK/MDR1). Therefore, **15k** may have the potential to inhibit MDR in certain cancer cells mediated by the overexpression of ABCB1 and/or ABCG2 transporters.

Finally, the safety of **15k** *in vivo* was determined in larval zebrafish model at different concentrations (0–100 μM). The zebrafish developed no signs of morphological or cardiac toxicities up to 10 μM of **15k**. In addition, **15k** did not significantly affect the swimming position of the larvae even at 10 μM after 48 h exposure (hpe). The zebrafish model is considered as very efficient model to predict drug toxicity in human including cardiotoxicity, neurotoxicity, developmental and gastrointestinal toxicity, among others (Barros et al., 2008; Eimon and Rubinstein, 2009). The larval stage allows for whole organism phenotypic toxicities detection and direct detection of organs functions (Westerfield, 1995). Previously, zebrafish was used to determine the toxicity of several anticancer agents. For example, sunitinib and axitinib killed 50% of zebrafish at concentrations 1.4 and 1 μM (LC_50_), with severe pericardial edema at 1 and 0.5 μM, respectively (Chimote et al., 2014). Here, we confirmed the safety of **15k** *in vivo* at relatively high concentrations. Further *in vivo* studies are needed to confirm the of **15k**.

## Conclusion

**15k** is a novel silybin derivative with significant cytotoxicity against OV2008 ovarian cancer cells. Compound **15k** significantly inhibited the formation rate of OV2008 colonies; induced apoptosis, reversed cell migration and invasiveness, and altered the expression of several proteins in the Wnt/β-catenin/EMT pathways (i.e., N-cadehrin, E-cadherin, c-Myc, DVL3, DVL2, and β-catenin). Interestingly, **15k** induced cleavage of Bax p21, yielding a novel, lower molecular weight fragment (Bax p18) that has potent apoptotic efficacy. Furthermore, **15k** induced the cleavage of β-catenin to lower molecular weight fragments that inhibited the transcriptional activity of β-catenin. **15k** reversed the mesenchymal phenotype in OV2008 cells and promoted the epithelial phenotype. Also, **15k** significantly decreased the expression level of the ABC transporters ABCB1 and ABCG2. Finally, **15k** showed good safety in larval zebrafish *in vivo* model after 48 hpe including cardiac, morphological and swim position parameters. Overall, our *in vitro* results indicate that **15k** has significant efficacy against OV2008 cells by affecting a number of biological targets. Future *in vivo* experiments will be conducted to determine its efficacy in animal.

## Author contributions

AT and HA: Setting up the idea of the research and setting up the experiments. HA, NH, CK, EM, AW, and CA: All participated in designing, running and analyzing the experiments. HA, AT, CA, PT, and TS: Writing, organizing providing critical input and reviewing the manuscript.

### Conflict of interest statement

The authors declare that the research was conducted in the absence of any commercial or financial relationships that could be construed as a potential conflict of interest.
